# Characterization of *RIN* Isoforms and Their Expression in Tomato Fruit Ripening

**DOI:** 10.3390/cells10071739

**Published:** 2021-07-09

**Authors:** Maria A. Slugina, Gleb I. Efremov, Anna V. Shchennikova, Elena Z. Kochieva

**Affiliations:** Institute of Bioengineering, Research Center of Biotechnology, Russian Academy of Sciences, Leninsky Ave. 33, bld. 2, 119071 Moscow, Russia; mashinmail@mail.ru (M.A.S.); gleb_efremov@mail.ru (G.I.E.); ekochieva@yandex.ru (E.Z.K.)

**Keywords:** tomato *Solanum lycopersicum* L., wild green-fruited tomato species, MADS-RIN, fruit ripening, gene structure, splicing, expression

## Abstract

Ripening of tomato fleshy fruit is coordinated by transcription factor RIN, which triggers ethylene and carotenoid biosynthesis, sugar accumulation, and cell wall modifications. In this study, we identified and characterized complete sequences of the *RIN* chromosomal locus in two tomato *Solanum lycopersicum* cultivars, its *rin/RIN* genotype, and three wild green-fruited species differing in fruit color and composition. The results reveal that *S*. *lycopersicum* cultivars and some wild species (*S. pennellii*, *S. habrochaites*, and *S. huaylasense*) had a 3′-splicing site enabling the transcription of *RIN1i* and *RIN2i* isoforms. The other wild species (*S. arcanum*, *S. chmielewskii*, *S. neorickii*, and *S. peruvianum*) had a 3′-splicing site only for *RIN2i*, which was consistent with *RIN1i* and *RIN2i* expression patterns. The genotype *rin*/*RIN*, which had an extended 3′-terminal deletion in the *rin* allele, mainly expressed the chimeric *RIN–MC* transcript, which was also found in cultivars (*RIN*/*RIN*). The RIN1, but not RIN2, protein is able to induce the transcription of the reporter gene in the Y2H system, which positively correlated with the transcription profile of *RIN1i* and RIN target genes. We suggest that during fruit ripening, RIN1 activates ripening-related genes, whereas RIN2 and RIN–MC act as modulators by competing for RIN-binding sites in gene promoters, which should be confirmed by further studies on the association between *RIN*-splicing mechanisms and tomato fruit ripening.

## 1. Introduction

Tomato *Solanum lycopersicum* L. is one of the most valuable crop plants in the world, producing fruit of high nutritional value. In addition, tomato is a good model to study the ripening of fleshy fruit, which is a complex process involving changes in color, texture, flavor, and taste [1]. The ripening process begins after the fruits have already reached their maximum size and transit from the mature green (MG) stage to fully heterotrophic stage, which is triggered by a climacteric boost in respiration and ethylene production [1]. At the molecular level, fruit ripening is regulated by three key transcription factors: MADS-domain RIPENING INHIBITOR (RIN or SlMADS-RIN), NAC NON-RIPENING (NOR), and SPL COLORLESS NON-RIPENING (CNR) [1,2]. Fruit-ripening regulation also includes transcription factors APETALA2a (AP2a), FRUITFULL (FUL1 and FUL2), and TOMATO AGAMOUS-LIKE1 (TAGL1) [2].

RIN is the main regulator of tomato fruit ripening. It controls the transcription of numerous genes involved in all stages of the process, including genes of the other two major transcription factors NOR and CNR, genes of the ethylene-dependent pathway, and structural genes associated with the ethylene-independent ripening network [3,4,5,6]. The list also includes genes responsible for the biosynthesis of carotenoids and aroma compounds, sucrose hydrolysis, and cell wall changes [3,4,5,6]. The accumulation of carotenoids, which define *S. lycopersicum* fruit color (yellow, orange, red, or brown), is already noticeable at the breaker (Br) stage and reaches a maximum at the red ripe (RR) stage [7]. In turn, carotenoids are converted to apocarotenoids, which serve as precursors for the synthesis of aroma compounds [8]. Sucrose hydrolysis and subsequent accumulation of monosaccharides define sweetness, whereas cell wall modifications—softness of ripe fruit [1].

*S. lycopersicum* belongs to the section Lycopersicon, which also includes 12 related wild tomato species mostly producing green fruit, except for *Solanum cheesmaniae* (L. Riley) Fosberg (yellow fruit), *Solanum galapagense* S.C. Darwin and Peralta (yellow-orange fruit), and *Solanum pimpinellifolium* B. Juss. (red fruit) [9,10]. The difference in fruit color between wild green-fruited (GF) tomatoes and *S. lycopersicum* cultivars is defined by carotenoid content [11], and that in taste by preferential accumulation of sucrose instead of glucose and fructose, respectively [12]. However, *S. lycopersicum* variants carrying the *rin* mutation are similar to GF species in fruit morphology and biochemical composition [2]. Thus, the fruit of both GF and *rin* plants retain their green color, do not accumulate moisture as they ripen, and remain firm for a longer time than cultivars. Such effects of the *rin* mutation on *S. lycopersicum* fruit are underlain by the suppression of cellular respiration and the associated ethylene release, which inhibits carotenoid accumulation, fruit softening, and production of aromatic compounds [2].

The *rin* mutation is caused by the deletion of a DNA region on chromosome 5, which results in the fusion of the adjacent truncated *RIN* and *MACROCALYX* (*MC*) genes (*RIN–MC*) [2,13]. The translated chimeric protein lacks a part of the RIN C-terminal activator domain and represents a gain-of-function mutant that actively represses ripening; in the nucleus, it interacts with other MADS-domain transcription factors and downregulates the expression of many ripening-related genes [13,14]. Therefore, it can be hypothesized that the differences in fruit ripening between wild GF tomato species and *S. lycopersicum* could be associated with the modifications of the *RIN* gene and its protein product. Understanding the role of the *RIN* and its isoforms in fruit ripening may be useful for breeding programs to increase fruit set quality.

In the current study, we tested this hypothesis by identifying and comparing complete sequences of the *RIN* chromosomal locus in wild-type *S. lycopersicum* cultivars, its *rin*/*RIN* genotype, and wild GF tomato species. We also performed expression profiling of the *RIN* isoforms in parallel with that of the RIN target genes involved in carotenoid and ethylene biosynthesis, sucrose hydrolysis, and cell wall metabolism, and tested the ability of the RIN1 and RIN2 isoforms to activate target gene transcription and interact with the known MADS-domain partners.

## 2. Materials and Methods

### 2.1. Plant Material

Seeds of *S. lycopersicum* cultivars, wild GF tomato species, and *S. lycopersicum rin*/*RIN* genotypes NN-25 and NN-21 (Table 1) were kindly provided by the N.I. Vavilov Institute of Plant Genetic Resources (VIR, St. Petersburg, Russia), the Federal Scientific Vegetable Center (FSVC, Moscow region, Russia), and the C.M. Rick Tomato Genetics Resource Center (TGRC, Davis, CA, USA). The NN-25 and NN-21 genotypes were progeny from the crossing of the cv. Zemba (VIR, St-Petersburg, Russia) with a line LA1795 homozygous for the *rin* mutation (unknown background; TGRC, Davis, CA, USA). Plants were grown in a greenhouse at 28/23 °C during the 16 h/8 h day/night light cycle (light intensity, 300–400 μmol m^−2^ s^−1^). Roots, leaves, flowers (anthesis), and fruit were collected in three biological replicates, homogenized in liquid nitrogen, and stored at −80 °C.

Fruits, forming red/red-to-violet (*S. lycopersicum* cultivars and NN-21 genotype) and pale yellow (NN-25 genotype) ripe fruits (Table 1), were harvested in planta at immature green (IG), MG, Br, and RR developmental stages. Fruits of wild GF tomato species were harvested in planta at MG and ripe final (RF) stages; fruit, both MG and RF, were green, of the final size, and hard and soft, respectively (i.e., the RR stage was defined by softness of the green fruit of a maximal size).

### 2.2. Identification and Structural Analysis of the RIN Locus

The sequences of the *RIN* chromosomal locus were amplified using genome-specific primers designed so that the neighboring amplicons overlapped (Supplementary Table S1). *RIN–MC* genomic sequences of *S. lycopersicum* cv. Heinz (Gene ID: 101252851) and *S. pennellii* (Gene ID: 107014532) were retrieved from NCBI GenBank (http://www.ncbi.nlm.nih.gov/; genome annotation releases (accessed on 1 November 2020)), and primers were manually designed considering sequence polymorphism and checked using Primer3 (http://frodo.wi.mit.edu/primer3/ (accessed on 11 August 2020)). Multiple sequence alignments were conducted with MEGA 7.0.26 [16]. RIN target gene-specific primers were designed based on the *S. lycopersicum* mRNA sequences available in NCBI GenBank: *PSY1* (NM_001247883.2), *PSY2* (NM_001247742.2), *Z-ISO* (NM_001347622.1), *ZDS* (NM_001247454.2), *PG2a* (NM_001247092.2), *EXP1* (NM_001247029.2), and *E4* (NM_001320202.1) (Supplementary Table S1).

Genomic DNA was isolated from young leaves of a single plant of each tomato accession as previously described [17] and used as a template (100 ng) for PCR amplification at the following conditions: initial denaturation at 94 °C for 5 min, 35 cycles of denaturation at 94 °C for 30 s, primer annealing at 55 °C for 30 s, extension at 65 °C for 2 min, and final extension at 65 °C for 5 min. The amplified PCR products of the expected size were purified by using the QIAEX^®^ II Gel Extraction kit (QIAGEN, Hilden, Germany), cloned in the pGEM^®^-T Easy (Promega, Madison, WI, USA), and sequenced (3–5 clones for each accession) on ABI Prism 377 DNA Sequencer (Applied Biosystems, Waltham, MA, USA) using the designed primers (Supplementary Table S1).

### 2.3. Gene Expression Analysis

Total RNA was extracted using the RNeasy Plant Mini Kit (QIAGEN, Hilden, Germany), purified from genomic DNA (RNase free DNasy set; QIAGEN, Hilden, Germany), qualified by gel electrophoresis, and used for first-strand cDNA synthesis (GoScript Reverse Transcription System; Promega, Madison, WI, USA) with an oligo-dT primer. RNA and cDNA concentrations were quantified by fluorimetry (Qubit^®^ Fluorometer, Thermo Fisher Scientific, Waltham, MA, USA).

Reverse transcription PCR was performed using 3.0 ng of cDNA and specific primers (Supplementary Table S1) at the following cycling conditions: initial denaturation at 95 °C for 5 min and 40 cycles of denaturation at 95 °C for 15 s and annealing/extension at 60 °C for 40 s. To confirm the amplification of the expected DNA fragment, PCR products were purified and sequenced. To ensure the specificity of the primers, a melting curve analysis was performed.

Quantitative (q) real-time (RT)-PCR was performed in 96-well plates with a CFX96 Real-Time PCR Detection System (Bio-Rad Laboratories, Hercules, USA), 3.0 ng of cDNA, SYBR Green RT-PCR mixture (Syntol, Moscow, Russia), and specific primers (Supplementary Table S1) at the following cycling conditions: initial denaturation at 95 °C for 5 min and 40 cycles of denaturation at 95 °C for 15 s and annealing/extension at 60 °C for 40 s.

To normalize the levels of gene expression, two reference tomato genes, *Expressed* (SGN-U346908) and *Actin 2/7* (NM_001330119.1) [18,19], were used. The qRT-PCR results were statistically analyzed with Graph Pad Prism version 8 (GraphPad Software Inc., San Diego, CA, USA; https://www.graphpad.com/scientific-software/prism/ (accessed on 1 March 2021)). The data were expressed as the mean ± standard deviation (SD) based on three technical replicates of three biological replicates for each combination of cDNA samples and primer pairs. The unequal variance (Welch’s) *t*-test was applied to assess the statistical significance (*p*-value < 0.05) of the differences in gene expression between samples.

### 2.4. In Vivo Analysis of Protein–Protein Interactions

Two-hybrid analysis was performed according to HybriZAP-2.1 Two-Hybrid cDNA Synthesis kit protocol (Stratagene, La Jolla, CA, USA) at room temperature and 30 °C, as previously described [20,21]. The bait and prey constructs were generated by cloning full-length cDNAs of the tomato genes into pAD-GAL4 and pBD-GAL4-Cam vectors (Stratagene, La Jolla, CA, USA).

### 2.5. Carotenoid Content

Total carotenoid content was measured by spectrophotometry in two biological and three technical replicates using a modified Folch method [22,23]. Briefly, 0.2 g of plant tissue was homogenized in Folch solution (chloroform:methanol 2:1 (*v*/*v*)) in the presence of trace Mg_2_CO_3_ amounts [23], incubated at 4 °C for 1 h, and centrifuged at 4000× *g* rpm for 10 min at 4 °C. The lower chloroform phase was collected and used for spectrophotometric assay of chlorophyll, lycopene, and β-carotene. Total carotenoid contents were measured in acetone–hexane solutions as previously described [24] using a spectrophotometer (Basic, Eppendorf, Hamburg, Germany) and quantified according to the following equations:

Chlorophyll a (µg/mL) = 11.47 (A666 − A750) − 2 (A648 − A750)

Chlorophyll b (µg/mL) = 21.85 (A648 − A750) − 4.53 (A666 − A750)

Total carotenoids (x + c) (µg/mL) = [1000 (A480 − A750) − 1.33 Chla − 23.93 Chlb]/202

Lycopene (mg/100 mL) = 0.204 A645 − 0.0458 A663 + 0.372 A505 − 0.0806 A453

β-Carotene (mg/100 mL) = 0.216 A663 − 1.22 A645 − 0.304 A505 + 0.452 A453

where A is the absorbance at the indicated wavelengths and x + c is the sum of xanthophylls and carotenes.

## 3. Results

### 3.1. Identification of the RIN Locus in the Analyzed Tomato Accessions

**Figure 1:**
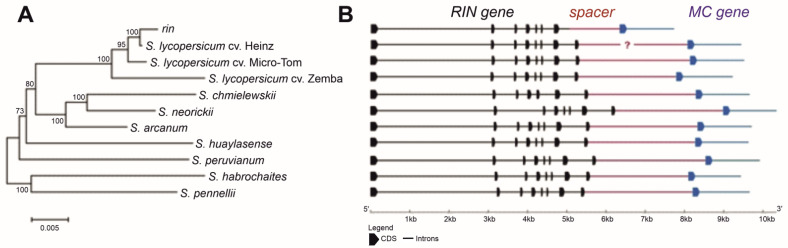
Phylogenetic relatedness and structure of the *RIN–MC* genomic region in *S. lycopersicum* cultivars, its *rin/RIN* genotype, and wild tomato species. (**A**) Phylogenetic tree. (**B**) The exon–intron structure. ?—the sequence is not defined; bars (black—for *RIN*; red—for spacer; blue—for *MC*) correspond to introns, blocks (black—for *RIN*; blue—for *MC*)—to exons.

The *RIN* genomic locus was identified (cloned and sequenced, or identified in silico) in three *S. lycopersicum* cultivars, its *rin*/*RIN* genotype, and seven wild GF tomato species (Table 1 and Table 2). The analyzed fragments included the complete *RIN* gene, *RIN–MC* intergenic spacer, and 5′-terminal fragment comprising exon I and partially intron I of the *MC* gene (Figure 1). The total length of this genomic region varied among the accessions: from 7724 bp in *rin*/*RIN* to 10,348 bp in *S. neorickii* (Figure 1).

All analyzed tomato accessions had the same structure of the *RIN* gene, which contained eight exons and seven introns; the gene length varied from 5288 (cv. Heinz) to 6230 bp (*S. neorickii*) (Figure 1 and Table 2). In the NN-25 genotype, both mutant (*rin*) and wild-type (*RIN*) alleles were identified, confirming the heterozygous state of the gene (*rin/RIN*); the *rin* allele (5089 bp) was characterized by the absence of the 3′-fragment of the intron VII and the whole exon VIII (Figure 1 and Table 2).

The level of *RIN* polymorphism among the analyzed tomato accessions was 10.43%: in total, 714 single nucleotide polymorphisms (SNPs) were identified; among them, 48 (0.7%) were found in *S. lycopersicum* cultivars and 638 (9.32%) in wild GF species. Three SNPs were specific to the NN-25 *rin* allele (compared to *S. lycopersicum* cultivars and wild GF species), 30 to wild GF species (compared to *S. lycopersicum* cultivars and NN-25) (Supplementary Figure S1), and none to *S. lycopersicum* cultivars compared to the *rin* mutant or wild GF species. Among exon SNPs, 14 were non-synonymous in wild species compared to cultivars; five amino acid substitutions were located in the conserved DNA-binding MADS domain, but none in the *RIN* gene of the analyzed GF species.

The analyzed accessions significantly differed in the *RIN–MC* spacer, whose length varied from 1395 (NN-25) to 2816 bp (cv. Micro-Tom) (Supplementary Figure S2). Despite good assembly and coverage of the *S. lycopersicum* cv. Heinz genome, it has an undefined region inside the *RIN–MC* spacer, which is also present in the genomes of other *S. lycopersicum* cultivars (such as M82 and I-3) available in the NCBI database. To determine the missing sequence, we tried to amplify it from the genomes of Heinz, All Round, Moneymaker, Ailsa Craig, and Yellow Peach cultivars using different primer pairs specific for the flanking sequences (Supplementary Table S1); however, there was no product, probably because of the presence of mobile elements or other extended sequences inside the *RIN–MC* spacer, which prevented its amplification. In other accessions analyzed in this study, the missing region was successfully amplified and sequenced (Supplementary Figure S2).

Thus, the structure of the analyzed *RIN* genomic fragment was similar in three *S. lycopersicum* cultivars and seven wild GF tomato species but differed from that of the *rin* allele. The *rin* locus had an extended deletion, which started in intron VII and included the entire exon VIII and a fragment of the *RIN–MC* intergenic spacer. The *RIN–MC* intergenic spacer was 1700 bp in cv. Micro-Tom, 1487 bp in cv. Zemba, 1491 bp in *S. habrochaites*, and 1673–1790 bp in the other wild GF species.

### 3.2. Identification of Splicing Sites in the RIN Homologs

**Figure 2:**
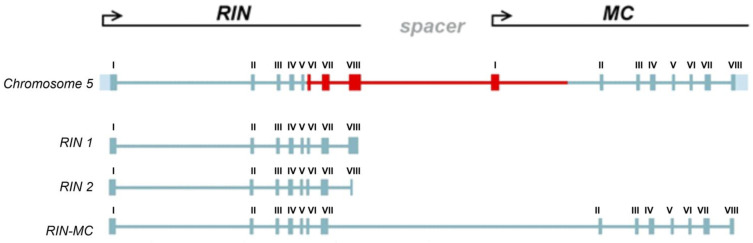
The *RIN–MC* genomic organization and exon composition of *RIN1i*, *RIN2i* and *RIN–MC* transcripts. Exons are indicated by boxes with the numbers above. The region of the locus subject to changes through *rin* mutations and gene splicing is colored red.

**Figure 3:**
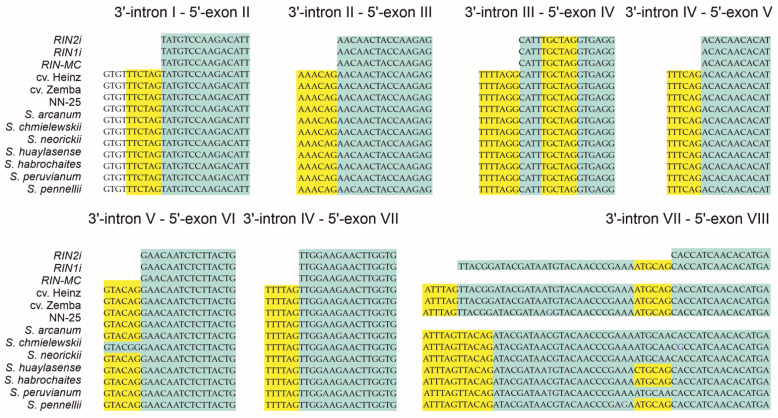
3′-splicing sites (highlighted yellow) identified in the introns of the *RIN* gene.

The *RIN* locus produces three different transcripts: isoforms *RIN1i* and *RIN2i*, and chimeric *RIN–MC*, which was previously identified in the *S. lycopersicum rin/rin* mutant [13] (Figure 2). All intron sequences of the identified *RIN* homologs contained a consensus of the main 3′-splicing site (NNNYAG) present in dicots [25] as well as an additional site TGCTAG at the beginning of exon IV; in *S. chmielewskii*, intron V 3′-splicing site contained an SNP (Figure 3). All GF species had a double 3′-splicing site in intron VII before exon VIII; another splicing site in this region was present in *S. lycopersicum*, *S. huaylasense*, *S. habrochaites*, and *S. pennellii* as well as in all *S. lycopersicum* cultivars (Figure 3). The NN-25 *rin* allele did not have the corresponding region of intron VII and exon VIII.

### 3.3. Expression of the Three RIN Transcripts in S. lycopersicum, rin/RIN, and Wild GF Species

To analyze the expression of different *RIN* transcripts, we used one common forward primer and three reverse primers specific for *RIN1i* (NM_001247741.2), *RIN2i* (NM_001315495.1), and *RIN–MC* (NM_001247047.2) (Supplementary Table S1), which allowed the amplification of full-length transcripts with the expected lengths of 729 (*RIN1i*), 660 (*RIN2i*), and 1194 bp (*RIN–MC*) (according to *S. lycopersicum* cv. Heinz *RIN–MC* sequence).

We determined the accumulation of the three transcripts in various organs of *S. lycopersicum* cv. Heinz, including roots, leaves, and flowers, as well as fruit pulp and skin at MG, Br, and RR developmental stages. The results reveal that *RIN1i* and *RIN2i* were expressed in all analyzed organs and tissues (except for the leaves which lacked *RIN2i* mRNA); the maximum level was observed in fruit, where it was increased at the Br and RR stages in the pulp and peel (Supplementary Figure S3). Surprisingly, the chimeric *RIN–MC* transcript was also found, albeit at a level much lower than those of *RIN1i* and *RIN2i*, in ripening fruit (Br and RR stages) (Supplementary Figure S3).

We compared the fruit-specific expression of *RIN1i*, *RIN2i*, and *RIN–MC* mRNA in *S. lycopersicum*, its *rin*/*RIN* genotype, and wild GF species. In the fruit of *S. lycopersicum* cv. Heinz and Zemba, the mRNA level of *RIN1i* was rather high, whereas that of *RIN2i* was lower; both isoforms were accumulated during fruit ripening, reaching a maximum at the Br and RR stages (Supplementary Figure S4). The *RIN–MC* transcript was expressed in fruit flesh of both cv. Zemba and Heinz at a level much lower than that of RIN1i and *RIN2i* (Supplementary Figure S4). In the *rin*/*RIN* genotype, the expression of *RIN1i* and *RIN2i* was weaker but still detected in all fruit stages (except for *RIN2i* at Br), thus confirming the heterozygous (*rin*/*RIN*) status of the *RIN* gene; the transcription of chimeric *RIN–MC* was first detected at the MG stage and was upregulated with fruit ripening, reaching a maximum at the RF stage (Supplementary Figure S4).

In the wild GF species *S. habrochaites* and *S. peruvianum*, the expression of *RIN1i* was observed in both hard and soft fruit of the final size (MG and RF, respectively), whereas that of *RIN2i* was much weaker and detected only in *S. habrochaites*, and that of *RIN–MC* was absent (Supplementary Figure S4). All the amplified PCR fragments were sequenced to confirm the amplification of the *RIN1i*, *RIN2i*, and *RIN–MC* transcripts (Supplementary Figures S5 and S6). The sequence of the *RIN–MC* transcript was the same for all analyzed accessions (Supplementary Figure S5). Semi-quantitative PCR allows only an approximate estimate of the levels of gene transcription, so we performed a quantitative analysis of gene expression.

### 3.4. RIN Isoform Expression Profiling in Developing Fruit of S. lycopersicum Cultivars, the rin/RIN Genotype, and Wild GF Species

**Figure 4:**
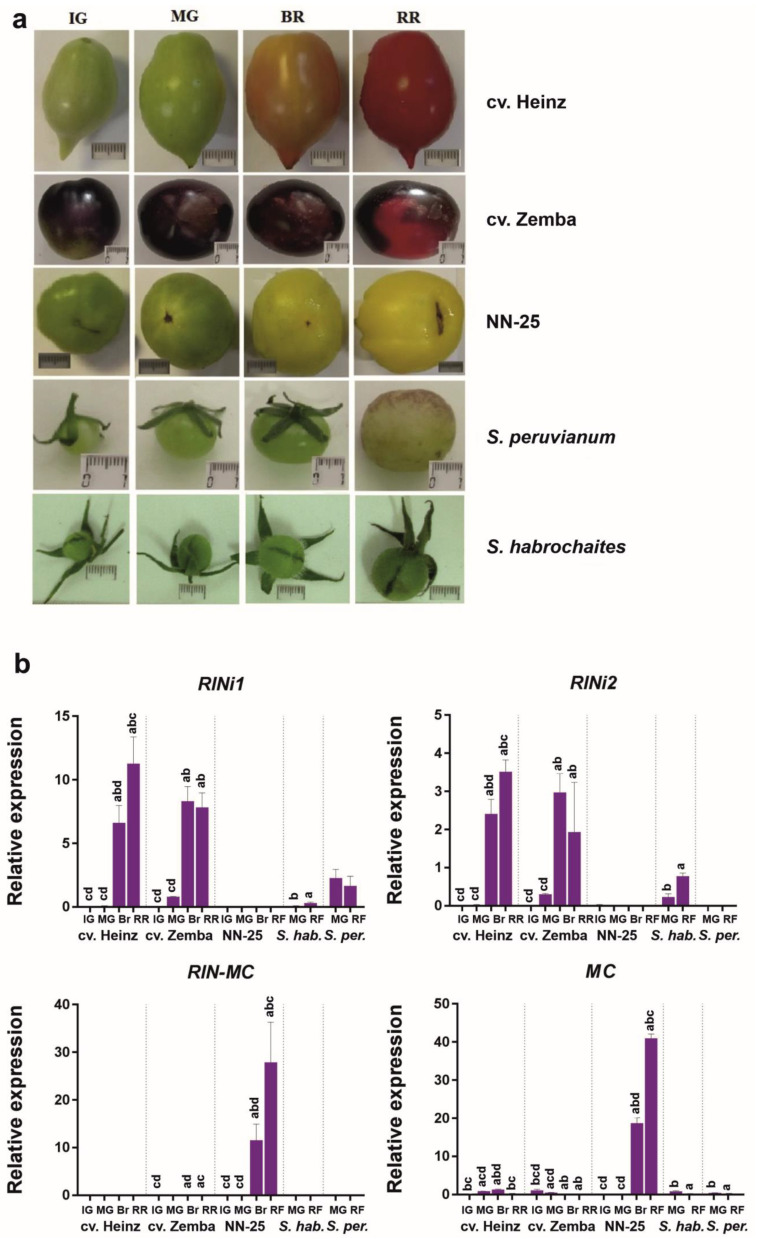
*RIN1i*, *RIN2i*, *RIN–MC*, and *MC* mRNA expression in the fruit of *Solanum lycopersicum,* its *rin*/*RIN* genotype, and wild tomato species. (**a**) Fruit images at different ripening stages; scale bar = 1 cm. (**b**) Quantitative expression analysis by qRT-PCR. NN-25, the *rin*/*RIN* genotype; *S. habr.*, *S. habrochaites*; *S. per.*, *S. peruvianum*; IG, immature green; MG, mature green; Br, breaker; RR, ripe red; RF, ripe final. Lowercase letters above the bars indicate statistically significant differences (*p* < 0.005) between gene expression in different tissues of the same accession: IG, a; MG, b; Br, c; RR (RF), d. For example, the gene expression in IG fruit was significantly different from that in other fruit, which is denoted by letters “bcd” above the bar.

Quantitative expression analysis by qRT-PCR confirmed that the fruit of *S. lycopersicum* cultivars had very weak expression of *RIN–MC* and quite strong expression of *RIN1i* and *RIN2i*; the mRNA levels of the *RIN1i* were almost three times higher than those of the *RIN2i* (Figure 4). The opposite expression pattern was revealed in the *rin*/*RIN* genotype: trace amounts of *RIN1i* and *RIN2i* (similar to RIN1i and RIN2i in cv. Zemba; Supplementary Table S2), and high amounts of RIN–MC mRNA. Between the analyzed GF species, the expression of *RIN1i* and *RIN2i* was detected in the fruit of *S. habrochaites*, although at a much lower level than in *S. lycopersicum*, whereas in those of *S. peruvianum*, only *RIN1i* was expressed (Figure 4), which corresponded to the absence of the splicing site required for *RIN2i* transcription (Figure 3). Except for the *rin*/*RIN* genotype, the expression of chimeric *RIN–MC* and *MC* mRNA (NM_001247736.1) was absent or negligible in all other species (Figure 4). In the *rin*/*RIN* genotype, *MC* mRNA significantly exceeds that in the other accessions, primarily due to the fact that the primer pair for MC is suitable for the *RIN–MC*; nevertheless, the difference between *MC* and *RIN–MC* levels suggests that the levels of *MC* transcription exceeds that in the other accessions by over 100 times (Figure 4). These results indicate that the transcriptional mechanisms underlying the expression of the *RIN* locus differed among wild GF species, *S. lycopersicum*, and its *rin*/*RIN* genotype. Considering that the *rin*/*RIN* plant should form red fruits due to the functional *RIN* allele, *CNR* gene expression was analyzed in mature green and ripe NN-25 and NN-21 fruits, and an almost 4–9-fold reduction in *CNR* transcription was found in NN-25 compared to NN-21 (Supplementary Figure S7).

### 3.5. Expression of RIN Target Genes during Tomato Fruit Development

**Figure 5:**
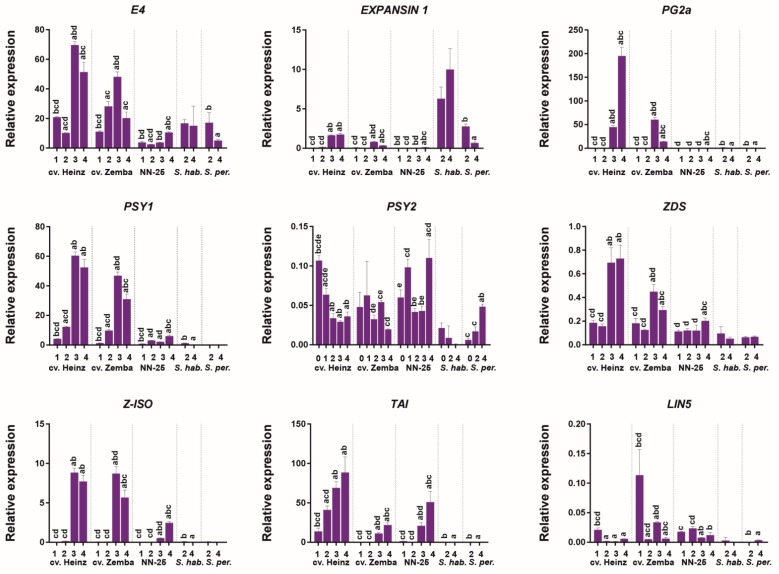
Expression of the RIN target genes during tomato fruit ripening in *Solanum lycopersicum* cultivars, its *rin*/*RIN* genotype, and wild GF tomato species. NN-25, the *rin*/*RIN* genotype; *S. hab.*, *S. habrochaites*; *S. per*., *S. peruvianum*; L, leaves (0); IG, immature green (1); MG, mature green (2); Br, breaker (3); RR, red ripe (or RF, red final) (4). Lowercase letters above the bars indicate statistically significant differences (*p* < 0.005) between gene expression levels in different tissues of the same accession: IG, a; MG, b; Br, c; RR (RF), d.

We analyzed the expression patterns of putative RIN target genes related to the fruit softening (*PG2a*, *EXPANSIN* 1 (*EXP1*), and *E4*), carotenoid biosynthesis (*PSY1*, *PSY2*, Z*-ISO*, and *ZDS*), and sucrose hydrolysis (*TAI* and *LIN5*). The expression of the *EXP1* gene was the strongest in GF species, weakest in *S. lycopersicum* cultivars, and absent in the NN-25, whereas that of the *E4* gene was the strongest in S. *lycopersicum* cultivars, weaker in GF species, and the weakest in the NN-25 (Figure 5). The expression of the *PG2a* gene was quite strong in Br and RR fruit of S. *lycopersicum* cultivars, but low in the other accessions (Figure 5).

The expression of *PSY1* in *S. lycopersicum* cultivars was upregulated during fruit ripening and was quite strong at its final stages, but very weak in the *rin*/*RIN* genotype, and almost absent in GF species (Figure 5). At the same time, the *PSY2* expression level was quite low both in *S. lycopersicum* cultivars and the *rin*/*RIN* genotype; it was the highest in the leaves of cv. Heinz and in RF of the *rin*/*RIN* genotype and the lowest in GF species (Figure 5). *Z-ISO* expression was detected only in Br and RR fruits of *S. lycopersicum* accessions; it was significantly stronger in wild-type plants than in the *rin*/*RIN* genotype (Figure 5). The expression of *ZDS* was found in all tested samples; it was the strongest in *S. lycopersicum* cultivars where it reached a maximum in Br and RR fruit, the weakest in *S. peruvianum*, and intermediate in *rin*/*RIN* where *ZDS* expression was slightly increased in RF fruit (Figure 5).

The expression of the *TAI* gene was detected in *S. lycopersicum* cultivars and *rin*/*RIN*, where it was upregulated with ripening, and was absent in *S. habrochaites* and *S. peruvianum*, whereas *LIN5* expression was very low in all analyzed accessions, especially in GF species (Figure 5).

### 3.6. Carotenoid Content in Ripe Fruit of S. lycopersicum Cultivars, Its rin/RIN Genotype, and GF Species

In RR fruit of S. lycopersicum cultivars, the carotenoid content (x + c, lycopene, and β-carotene) was equally high, whereas in ripe fruit of the rin/RIN and GF species the total carotenoid (x + c) content was about 10 times lower, the level of β-carotene was similar (S. peruvianum) or 2–7 times lower (NN-25 and S. habrochaites), and lycopene was absent (Table 3).

### 3.7. Analysis of Protein–Protein Interactions of RIN1 and RIN2 Isoforms

The interactions of RIN1 and RIN2 proteins with the known RIN partners, the MADS-domain transcription factors FRUITFULL 2 (FUL2) and TOMATO AGAMOUS-LIKE 1 (TAGL1), were analyzed in vivo in yeast cells. The results indicate that both isoforms could interact with the two proteins, but such interactions were much stronger for RIN1 than for RIN2 and only RIN1 demonstrated the ability to activate the transcription of the reporter gene, whereas RIN2 did not (Table 4).

## 4. Discussion

The transcription factor RIN plays an important role in the regulation of the tomato fruit ripening; it does not participate in ripening induction but is strictly necessary for full ripening and prevention of over-ripening [1,2,3,4,5,6,27]. RIN activates gene transcription through its C-terminal domain and could also interact with different MADS-domain proteins through its other regions, forming multimeric complexes that bind to the promoters of target genes [27]. In the *rin* mutant, the *RIN* gene lacks exon VIII and part of exon VII (corresponding to the C-terminus) and is inactivated [27].

In the present study, we identified the RIN locus in S. lycopersicum cultivars, its rin/RIN genotype, and wild GF tomato species significantly differing in the morphology, taste and color of ripe fruit, which are red and soft in cultivars, green and soft in wild species, and pale yellow and firm in the rin/RIN genotype; such distictions depend on carotenoid accumulation, sugar content, and ethylene biosynthesis. Pale yellow ripe fruits of the NN-25 genotype are close in color to those of wild species, whereas the rin/RIN ripe fruits are reported to be red [28]. A significant decrease in CNR expression was observed in fruits of NN-25 compared to NN-21 (the same background as in NN-25) (Supplementary Figure S7). The Cnr allele is epistatic over the rin allele and masks the rin effects [28], and Cnr epimutation in the promoter is accompanied by a sharp decrease in CNR expression level and pale yellow fruit phenotype [29]. Given this, we may assume that the NN-25 fruits have a pale yellow color due to either the possible rin/RIN Cnr/CNR genotype, or very low (in comparison with red-fruited tomatoes) PSY1 gene expression (Figure 5).

In terms of ripening characteristics, the fruit of the NN-25 are closer to those of wild tomato species than to those of cultivars despite the fact that the *rin* locus of the NN-25 has a characteristic deletion [2], whereas the *RIN* structure in GF species is more similar to that of cultivars (Figure 1, Supplementary Figure S1). Thus, it can be suggested that the incomplete fruit ripening in wild GF tomato species and *rin*/*RIN* is caused by different mechanisms.

**Figure 6:**
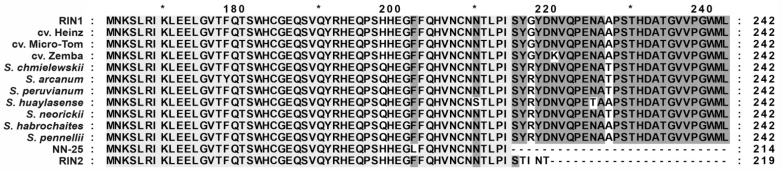
Alignment of the C-termini of RIN1 isoforms from *S. lycopersicum* cultivars, and wild GF species. NN-25, the product of the NN-25 *rin* allele; RIN2, *S. lycopersicum* cv. Heinz RIN2 isoform.

The *S. lycopersicum RIN* gene produces two mRNA isoforms (*RIN1i* and *RIN2i*) because of the presence of the alternative 3′-splice site; the respective proteins RIN1 (NP_001234670.1) and RIN2 (NP_001302424.1) differ in the C-terminus (Figure 2 and Figure 6) and the longer variant, RIN1, has been reported to act as a transcriptional activator [28,29,30,31,32,33,34]. The *rin*/*rin* mutant produces the *RIN–MC* fusion transcript, but the transcriptional activation potential of the RIN–MC protein is controversial: although it has been shown to induce the luciferase (*LUC*) gene, it rather does not activate the ripening genes [13,14].

The ability of RIN to perform transcriptional activation depends on its C-terminus [35], which in RIN2 is five residues longer (TLPISTINT) than in the RIN of the mutant *rin* allele or RIN–MC (TLPI) (Figure 6, Supplementary Figure S5), suggesting that, since RIN–MC may perform transcriptional activation, RIN2 could also do so through binding to the RIN target genes. However, our analysis of the RIN1 and RIN2 in a two-hybrid system indicated that RIN2 could dimerize with TAGL1 and FUL2 (Table 4), known as RIN partners [30,31,32,33,34], but could not activate the transcription of the reporter gene. Given these results and the fact that MC does not act as a transcriptional activator [35], the RIN–MC protein should also lack the ability to induce transcription.

Overall, these findings suggest that RIN1 activates fruit ripening-related genes, whereas RIN2 and RIN–MC regulate the RIN target genes by competing with RIN1 for the binding sites. It is possible that the plant has developed such a system for high-precision tuning of the fruit-ripening process.

The immaturity of the *rin* fruit is associated with the expression of *RIN–MC* and the absence of that of *RIN (RIN1)* [2,13,14]. This is consistent with our results showing that the identified *S. lycopersicum RIN* homologs could produce a significant amount of the *RIN1i* transcript, whereas the NN-25—only its traces; moreover, the NN-25 highly expresses the *RIN–MC* (Figure 4). At the same time, the *RIN* genes of GF species are split in terms of the presence of the splicing site for the *RIN2i* transcript: some of them had it (*S. pennellii*, *S. habrochaites*, and *S. huaylasense*) and some did not (*S. arcanum*, *S. chmielewskii*, *S. neorickii*, and *S. peruvianum*) (Figure 3). This is consistent with qRT-PCR results showing the low presence of *RIN1i* in both *S. habrochaites* and *S. peruvianum* fruits, and *RIN2i* only in *S. habrochaites* fruits (Figure 4). In *S. habrochaites*, the RIN2 may compete with RIN1 for target ripening genes, suppressing their expression, which may explain the formation of green-colored fruits. The green fruits of *S. peruvianum* are likely to be due to the fact that the expression of *RIN1i* is about 4–5 times lower than that of the *S. lycopersicum* cultivars (Figure 4).

Expression analysis confirmed that all three *RIN* transcripts were expressed in *S. lycopersicum* cultivars; however, the *RIN2i* expression level was 2–3 times lower than that of *RIN1i* and only trace amounts of the *RIN–MC* transcript were detected (Supplementary Figures S3 and S4, Figure 4). It should be noted that *RIN–MC* expression has not been previously reported in wild-type tomatoes but only in *rin* mutants [13]. It can be hypothesized that the *RIN–MC* transcript is expressed in *S. lycopersicum* accessions because of the presence of 3′-splicing site AAACAG upstream of the *MC* sequence involved in the fusion (Supplementary Figure S5). However, the *MC* genes of GF species *S. peruvianum* (KY471421.1) and *S. habrochaites* (KY471429.1) also had this 3′-splicing site but did not express *RIN–MC* mRNA (Supplementary Figure S4, Figure S5). Possibly, small amounts of the *RIN–MC* transcripts are normally synthesized at the *RIN–MC* locus using existing splice sites, whereas in the mutant *rin* allele, due to the deletion of the last exon of the *RIN* gene, the equilibrium is shifted towards the formation of *RIN–MC*.

In the *rin*/*RIN* genotype, the transcription levels of *RIN1i* and *RIN2i* were significantly lower, whereas those of chimeric *RIN–MC* were significantly higher than in the cultivars (Figure 5 and Figure 6), which could be due to the presence of only one wild-type allele and the absence of splice sites for *RIN1i* and *RIN2i* in the *rin* allele. The expression of both splicing variants in GF *S. habrochaites* and only *RIN1i* in *S. peruvianum* (Figure 5 and Figure 6; Supplementary Figure S1) was in accordance with the presence of the *RIN2i* splicing site in the *S. peruvianum RIN* and its absence in the *S. habrochaites RIN* (Figure 3).

RIN binds to over a thousand genomic regions, including promoters of numerous ripening-related genes, including those encoding transcription factors and proteins involved in ethylene production, carotenoid biosynthesis, and cell wall modification [3,4,5,35,36,37]. The RIN-exerted effects can be either transcriptional upregulation (RIN1), downregulation (RIN–MC), or more complex modulation including both RIN1 and RIN–MC [13], which is consistent with our results. Therefore, the differential transcription of the RIN isoforms in the analyzed tomato accessions should directly affect the expression of the target genes. Significantly lower *RIN1i* mRNA levels in GF species and *rin*/*RIN* may be correlated with the decreased production of carotenoids (Table 3) as well as ethylene biosynthesis, which should change the color and increase the hardness of the fruit, respectively [6]. Genes involved in the biosynthesis of carotenoids are differentially regulated by RIN isoforms. For example, the expression of the phytoene synthase gene *PSY1* required for phytoene/lycopene synthesis during tomato ripening can be regulated by both RIN and RIN–MC, whereas that of *PSY2* involved in the synthesis of carotenoids in immature green fruit is regulated by RIN–MC [3,13]*. Z-ISO*, which encodes carotene zeta isomerase converting colorless 15-cis phytoene to red trans-lycopene [38], is regulated by RIN and RIN–MC [3,13], whereas *ZDS* encoding zeta-carotene desaturase, which converts zeta-carotene to lycopene, is regulated by RIN but not RIN–MC [13]. Our results indicate that in the analyzed tomato accessions, the expression level of *RIN1i* (Supplementary Figures S3 and S4, Figure 4) was directly correlated with those of *PSY1*, *Z-ISO*, and *ZDS* (Figure 5) and carotenoid content (Table 3): all were high in red fruit of *S. lycopersicum* cultivars and low in pale yellow and green fruit of the *rin/RIN* and GF species, respectively*,* which is consistent with *PSY1* expression levels in cultivars and the *rin*/*RIN Cnr*/*CNR* revealed in a previous study [28]. The expression of PSY2, which is mostly observed in chloroplasts, was similar in the leaves and fruit (Figure 5), suggesting that the chloroplast-specific carotenoid synthesis was maintained at the basic low level in both photosynthetic and reproductive tissues of the analyzed accessions and, furthermore, that the RIN–MC did not activate the *PSY2* gene as has been proposed earlier [13].

The other RIN target genes, *TAI* and *LIN5* [13,39], are responsible for the taste and texture of tomato fruit. *TAI* encodes vacuolar invertase, converting sucrose, which is accumulated in wild GF tomato species, to glucose and fructose, characteristic for ripe fruit of *S. lycopersicum* cultivars [12], whereas *LIN5* encodes cell wall invertase, which affects the biosynthesis of cuticle components and sugar absorption by tomato fruit [40]. In this study, the expression of *TAI* and *LIN5* was noticeable only in *S. lycopersicum* (cultivars and NN-25) (Figure 5) and corresponded to high hexose content in their fruit (Table 1). However, there was no correlation between *TAI*/*LIN5* and *RIN1i* mRNA levels, suggesting that *TAI* and *LIN5* may be competitively regulated by RIN1 and RIN2/RIN–MC, which exert positive and negative, respectively, transcriptional effects. A low level of *LIN5* expression may indicate that this gene is involved in the control of sugar content only in the cuticle but not in the whole fruit.

The PG2a gene encodes polygalacturonase-2a associated with cell wall modification (polyuronide degradation) and texture. PG2a expression, which is induced by ethylene and reaches a maximum in ripe fruits [41,42], has been shown to be regulated by RIN–MC but not RIN (RIN1) [13], although the presence of RIN-binding sites in the promoter [3] implies that it should respond to all RIN isoforms. We observed strong PG2a expression only in Br and RR fruit of S. lycopersicum cultivars (Figure 5), suggesting that RIN1, rather than RIN–MC, may be involved in PG2a upregulation.

EXP1, which is one of the key genes defining fruit softness [43], is suggested to be regulated by both RIN (RIN1) and RIN–MC [13]. However, the RIN1 and RIN–MC expression profiles (Supplementary Figure S4, Figure 4) suggest that EXP1 transcription (Figure 5) can be repressed by RIN–MC.

The *E4* gene encoding methionine sulfoxide reductase, a repair enzyme for oxidation-damaged proteins, is a target of RIN1 but not RIN–MC [13] and is upregulated after ethylene induction [44]. In this study, the *E4* transcription levels were high in cultivars, intermediate in GF species, and low in the *rin*/*Rin* genotype (Figure 5), and were correlated with *RIN1* expression (Figure 4), confirming the role of RIN1 in *E4* activation.

## 5. Conclusions

In this study, we identified and characterized complete sequences of the *RIN* chromosomal locus in *S. lycopersicum* cultivars, its *rin*/*RIN* genotype, and wild GF tomato species, which differ in ripe fruit morphology and composition. Cultivars, as well as some wild species (*S. pennellii*, *S. habrochaites*, and *S. huaylasense*), had a splicing site in intron VII/exon VIII of the *RIN* gene, which enabled the transcription of two *RIN* isoforms, *RIN1i* and *RIN2i*, whereas the other GF species (*S. arcanum*, *S. chmielewskii*, *S. neorickii*, and *S. peruvianum*) did not have this splicing site. Accordingly, *S. lycopersicum* cultivars as well as GF *S. habrochaites* expressed *RIN1i* and *RIN2i*, whereas *S. peruvianum—*only *RIN1i*. The *rin*/*RIN* genotype had an extended deletion, including exon VIII and a fragment of the *RIN–MC* intergenic spacer, and mostly expressed the chimeric *RIN–MC* fragment, which was also detected in *S. lycopersicum* cultivars. Although both RIN1 and RIN2 could interact with MADS-domain-containing RIN partners, only RIN1 could activate the transcription of reporter genes, which was consistent with the positive correlation between the expression of putative RIN target genes and that of *RIN1i*. We assume the existence of a link between differences in fruit ripening of wild GF tomato species and *S. lycopersicum* cultivars with modifications of the *RIN* gene and its protein product. High expression of the functional *RIN* isoform, *RIN1*, which prevails over the expression of *RIN2i* and *RIN–MC*, in fruits of tomato cultivars leads to the activation of ripening genes that are RIN targets; as a result, the fruit turns red (or yellow, pink, brown, etc.*—*due to the accumulation of carotenoids, mainly lycopene). The extremely low expression of *RIN1i* and the high level of *RIN–MC* in the *rin/RIN* genotype correspond to a pale yellow color of the fruit due to the low presence of the RIN1 protein (competing with RIN–MC for partners and targets), which leads to a weak induction of the expression of carotenoid pathway genes, *PSY1*, *ZDS*, etc. Low amounts of *RIN1i*, *PSY1*, *ZDS*, and *Z-ISO* transcripts, as well as the presence of *RIN2i* and *RIN–MC* in wild tomato species, correspond to ripe green fruits without lycopene. These results provide further insights into the molecular mechanisms regulating tomato fruit ripening.

## Figures and Tables

**Table 1:** Characteristics of the analyzed tomato accessions.

Accession	Catalogue Number	Ripe Fruit Color	Primary Storage Sugars
*S. lycopersicum* cv. Micro-Tom	LA3911	Red	Glucose, fructose [15]
*S. lycopersicum* cv. Heinz 1706-BG	LA4345	Red	Glucose, fructose [15]
*S. lycopersicum* cv. Zemba	FSVC 11277	Red to violet	Glucose, fructose [15]
*rin*/*RIN*	FSVC NN-25	Pale yellow	Glucose, fructose ^1^
*RIN*/*RIN*	FSVC NN-21	Red	Glucose, fructose [15]
*S. chmielewskii*	LA 2663	Green	Sucrose [15]
*S. neorickii*	LA1326	Green	n/a
*S. arcanum*	LA2157	Green	n/a
*S. huaylasense*	LA1365	Green	Sucrose [15]
*S. peruvianum*	VIRR 4361	Green	Sucrose [15]
*S. habrochaites*	LYC4	Green	Sucrose [15]
*S. pennellii*	LA0716	Green	n/a

^1^ This study.

**Table 2:** Characteristics of the *RIN–MC* locus in the analyzed tomato accessions.

Species	*RIN*		*RIN1i*			*RIN2i*			*RIN–MC*			Intergenic Spacer bp
	Accession Number	Total bp	Transcript bp	Accession Number	Protein aa	Transcript bp	Accession Number	Protein aa	Transcript bp	Protein aa	Accession Number	
*S. lycopersicum* cv. Micro-Tom	CM022786.1 (1792…7111) ^1^	5320	729	n/a	242	660	n/a	219	1194	397	n/a	2816
*S. lycopersicum* cv. Heinz 1706	NC_015442.3 (2081…7368) ^1^	5288	729	MW889896	242	660	MW889900	219	1194	397	MW889904	n/d
*S. lycopersicum* cv. Zemba	MW889907	5291	729	MW889897	242	660	MW889901	219	1194	397	MW889905	2511
*rin/RIN* genotype NN-25	MW889911	5089	RIN exon VIII is absent in the *rin* allele						1194	397	MW889906	1395
*S. chmielewskii* LA 2663	MT228441	5543	729	n/a	n/a	660	n/a	219	n/a	n/a	n/a	2741
*S. neorickii* LA1326	MW889908	6230	729	n/a	n/a	660	n/a	219	n/a	n/a	n/a	2749
*S. arcanum* LA2157	CBYQ010009972.1 (20826…21107); CBYQ010009973.1 (288...5582) ^1^	5582	729	n/a	n/a	660	n/a	219	n/a	n/a	n/a	2748
*S. huaylasense* LA1365	MW889909	5536	729	n/a	n/a	660	n/a	219	n/a	n/a	n/a	2734
*S. peruvianum* LA4361	MW889910	5742	729	MW889898	242	660	n/d	219	1194	397	n/d	2116
*S. habrochaites* LYC4	CBYS010023633.1 (12571…18159) ^1^	5589	729	MW889899	242	660	MW889902	219	1194	397	n/d	2505
*S. pennellii* LA0716	CCXL01022058.1 (2300…7750) ^1^	5451	729	MW928510	242	660	MW889903	219	n/a	n/a	n/d	2754

^1^ Sequences were extracted from the WGS NCBI database; n/d—not detected, n/a—not analyzed.

**Table 3:** Carotenoid content in the analyzed tomato accessions.

Accession	Carotenoid Content in Ripe Fruits (mg/g Wet Weight)		
	Total (x + c)	Lycopene	β-Carotene
*S. lycopersicum* cv. Heinz 1706-BG	0.2197 ± 0.0398	0.1592 ± 0.0386	0.0212 ± 0.0037
*S. lycopersicum* cv. Zemba	0.2701 ± 0.0822	0.1488 ± 0.0466	0.0219 ± 0.034
*rin*/*RIN*	0.0047 ± 0.0011	0.0055 ± 0.0027	0.0033 ± 0.0013
*S. peruvianum*	0.0195 ± 0.0018	0	0.0242 ± 0.0045
*S. habrochaites*	0.0189 ± 0.0065	0	0.0099 ± 0.0034

**Table 4:** Protein–protein interactions of RIN1 and RIN2 isoforms ^1^.

pAD_GAL4	pBD_GAL4cam	˗LH +10 mM 3AT	˗LTH +10 mM 3AT	˗LTA	X-gal Test
Autoactivation test					
	RIN2	−		−	−
	RIN1	++		+	+
	CDM44 (+control)	+		+	+
	CDM37 (−control)	−		−	−
Protein–protein interaction test					
RIN2	TAGL1		+	+ −	−
RIN2	FUL2		+	+ −	−
RIN1	TAGL1		++	++	++
RIN1	FUL2		++	++	++
CDM44 (+control)	CDM37 (+control)		+	+	+
CDM44 (−control)	CDM111 (−control)		−	−	−

^1^ The experiment was carried out both at room temperature and 30 °C and the same results were obtained at both temperatures. L—L-leucine, H—L-histidine, T—L-tryptophan, A—L-adenine hemisulfate salt, 3AT—3-amino-1,2,4-triazole, ˗LH, ˗LTH and ˗LTA—medium devoid of these amino acids, X-gal—5-bromo-4-chloro-3-indolyl-β-d-galactopyranoside. *Chrysanthemum* MADS-domain proteins CDM44, CDM37, and CDM111 were used as controls [26].

## Data Availability

Sequences of the *RIN* locus and the *RIN1*, *RIN2* and *RIN-MC* transcripts were deposited in the NCBI database (listed in Table 2).

## Supplementary Materials

The following are available online at https://www.mdpi.com/article/10.3390/cells10071739/s1, Figure S1: Alignment of the *RIN* gene from *S. lycopersicum* cultivars, *rin/RIN* genotype (*rin* allele), and wild GF tomato species; Figure S2: Alignment of the spacer between *RIN* and *MC* genes from *S. lycopersicum* cultivars, *rin/RIN* genotype *(rin* allele), and wild GF species; Figure S3: Expression of *RIN1i*, *RIN2i*, and *RIN–MC* in different organs and tissues of *S. lycopersicum* cv. Heinz. R, roots; L, leaves; F, opened flowers; MG, mature; Figure S4: Fruit-specific expression of *RIN1i*, *RIN2i*, and *RIN–MC*. NN-25, the *rin*/*RIN* genotype; *S. hab*., *S. habrochaites*; *S. per*., *S. peruvianum*; IG, immature green (flesh); MG, mature green (flesh); Br, breaker (flesh); RR, ripe red (flesh); RF, ripe final (flesh). The *Actin* gene was used as reference; Figure S5: Alignment of the *RIN–MC* transcript from *S. lycopersicum* cultivars and the *rin/RIN* genotype; Figure S6: Alignment of the *RIN1i* and *RIN2i* transcripts from *S. lycopersicum* cultivars, *rin*/*RIN* genotype, and wild tomato species; Figure S7: Expression of the *CNR* gene in mature green and ripe fruits of the NN-21 and NN-25 accessions. Lowercase letters above the bars indicate statistically significant differences (*p* < 0.005) between gene expression levels; Table S1: List of primers used for gene amplification, sequencing, and expression analysis; Table S2: *RIN1i*, *RIN2i*, and *RIN–MC* relative expression data in fruits of *S. lycopersicum* cultivars, *rin*/*RIN* genotype NN-25, and wild GF tomato species.
